# Rheological Behaviors of Rubber-Modified Asphalt Under Complicated Environment

**DOI:** 10.3390/polym17131753

**Published:** 2025-06-25

**Authors:** Xia Wu, Chunfeng Zhu, Zhenyu Wang, Lei Yang, Fang Liu, Jianxin Chen, Khusniddin Nuriddinov, Shukhrat Giyasov, Natalia Borisovna Morozova, Wenqing Shi, Chao Lu, Anastassios Papageorgiou, Di Tie

**Affiliations:** 1 College of Civil Engineering, Jilin Jianzhu University, Changchun 130118, China; 2 School of Materials Science and Engineering, Northeastern University, Shenyang 110819, China; 3 School of Materials Science and Engineering, Guangdong Ocean University, Zhanjiang 524000, China; 4 Faculty of Science, University of Turku, FI-20014 Turku, Finland

**Keywords:** crumb rubber powder, rubber-modified asphalt, de-icing salt erosion, microstructural evolution, viscoelastic behavior

## Abstract

While crumb rubber powder has demonstrated effectiveness in enhancing the mechanical properties of asphalt binders, its viscoelastic behavior under freeze–thaw conditions in clean water and de-icing salt, typically urban road conditions in winter, remains insufficiently explored. This study systematically investigated the microstructural evolution, compositional changes, and mechanical behavior of asphalt modified with rubber under the influence of freeze–thaw conditions in clean water and de-icing salt. The findings revealed that rubber powder incorporation accelerates the precipitation of oil, enhancing material stability in both aqueous and saline environments. Notably, asphalt containing 10% crumb rubber powder (Asphalt-10% RP) and 20% crumb rubber powder (Asphalt-20% RP) exhibit creep recovery rates 50.53% and 28.94% higher, respectively, under de-icing salt freeze–thaw cycles than under clean water freeze–thaw cycles. Therefore, in regions with extremely low temperatures and frequent snowfall, rubber powder exhibits significant research potential, providing theoretical support for the design of asphalt pavements in cold climates.

## 1. Introduction

Arctic and alpine regions face severe road icing issues due to extreme cold and frequent snowfall. These conditions significantly increase traffic accident risks [1,2,3] and related fatalities [4]. Annual consumption of de-icing salt surpasses 2 million metric tons to maintain winter road safety. However, prolonged icing exposure and chemical de-icing salt accelerate the performance degradation of asphalt pavements [5,6,7,8,9]. There is therefore an urgent requirement to engineer advanced asphalt materials that simultaneously resist freeze–thaw damage and de-icing salt corrosion.

As a resource-rich, renewable, and environmentally friendly material, rubber powder has demonstrated remarkable potential in harsh environments. Previous research has revealed that the fatigue resistance of an asphalt mixture can be effectively improved by adding diatomite and basalt fiber [10]. In addition, basalt fibers and polyester fibers incorporated into plant-mixed hot recycled asphalt mixes enhance their mechanical properties, but basalt fibers show a more pronounced enhancement of the properties of recycled asphalt mixes [11]. Notably, after modification, although numerous additives can improve the mechanical properties of asphalt mixtures, rubber-modified asphalt pavements not only enhance cold-region road performance but also promote sustainable infrastructure development [12,13,14]. Khasawneh et al. [12] demonstrated that crumb rubber particles, upon incorporation into asphalt binders, absorb maltenes and swell, thereby increasing binder viscosity and improving stiffness. Shu et al. [15] also demonstrated that incorporating crumb rubber into asphalt improves mixture performance by enhancing binder mechanical properties of the asphalt binder. These performance enhancements arise from combined physical adsorption and chemical crosslinking mechanisms that restructure the binder’s microstructure, thereby enhancing the asphalt’s mechanical properties [16,17]. However, while existing research has thoroughly examined the mechanical properties of crumb rubber-modified asphalt mixtures under normal ambient temperature and humidity conditions, investigations into their long-term performance under sustained icing and de-icing salt exposure remain scarce.

In recent years, the annual application of de-icing salt on cold-region roads has been increasing. The early damage of asphalt pavements caused by the coupled action of salt corrosion and freeze–thaw cycles has become a prominent research focus in road engineering. Investigations in salt lake areas, saline soils, and regions with frequent de-icing agent use have shown that salts such as NaCl and Na_2_SO_4_ infiltrate asphalt mixtures through pavement voids. This infiltration significantly reduces the adhesiveness of asphalt and the bond between asphalt and aggregates, thereby inducing diseases like aggregate stripping and pavement pitting [18,19]. Therefore, revealing the degradation mechanism of asphalt materials in salt-corrosive environments and proposing targeted improvement strategies are of critical importance for the durability design of cold-region pavements. Early studies focused on the impact of salt solutions on the macroscopic mechanical behavior of asphalt mixtures. Goh and Xiong et al. [20,21] conducted a series of laboratory mechanical tests. They found that the mechanical properties of asphalt mixtures treated with salt solutions degraded to varying degrees. Recent research has deepened our understanding of the micro-degradation mechanisms of asphalt induced by salt corrosion. Multiple studies have indicated that salt solution erosion promotes a key transformation: light components in asphalt, including saturated hydrocarbons and aromatics, are converted into heavy components such as asphaltenes. This transformation is accompanied by significant increases in oxygenated functional groups, including sulfoxide (S=O) and carbonyl (C=O) groups [22,23,24,25]. These chemical changes directly affect the physicochemical properties of asphalt. However, existing studies mainly characterize and interpret the mechanical property decline caused by salt corrosion and its microscale causes. Research on effectively enhancing the durability of asphalt materials in de-icing salt environments remains scarce. In response, this study focuses on a promising waste crumb-rubber modifier. It systematically explores the modifier’s improvement effects and mechanisms on the performance of asphalt binders under de-icing salt erosion. The findings aim to provide theoretical support for subsequent pavement design in cold regions.

This study systematically evaluates the influence of freeze–thaw cycles in water and de-icing salt solutions on rubber-modified asphalt with diverse crumb rubber contents. Optical microscopy (OM), atomic force microscopy (AFM), and Fourier-transform infrared (FTIR) spectroscopy were applied to characterize microstructural evolution and chemical compositional changes. The multiple stress creep recovery (MSCR) and linear amplitude sweep (LAS) experiments characterized the rheological behavior of rubber-modified asphalt under different harsh conditions. Through comprehensive evaluation of microstructural, chemical, and mechanical responses, this investigation provides critical insights for developing deicer-resistant asphalt pavements in cold regions.

## 2. Materials and Methods

### 2.1. Materials Preparation

**Figure 1:**
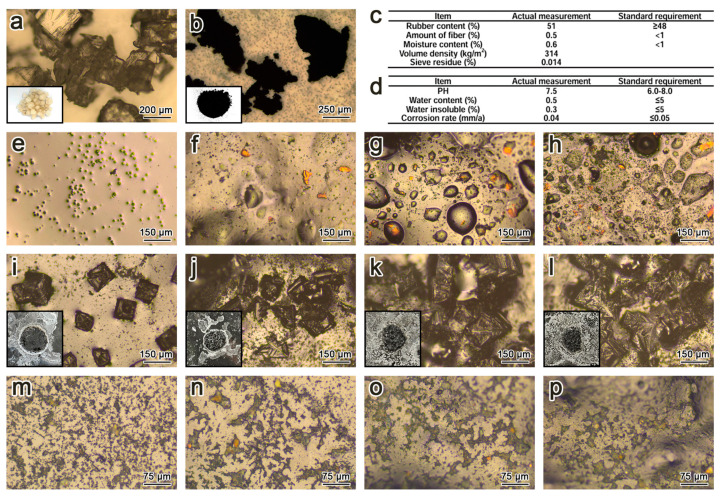
(**a**) Image of de−icing salt. (**b**) Image of rubber powder. (**c**) Basic indexes of rubber power. (**d**) Basic indexes of de-icing salt. (**e**) Matrix asphalt (CW). (**f**) Asphalt−10% RP (CW). (**g**) Asphalt−20% RP (CW). (**h**) Asphalt−30% RP (CW). De-ionized water before rinsing: (**i**) matrix asphalt (SA); (**j**) Asphalt−10% RP (SA); (**k**) Asphalt−20% RP (SA); (**l**) Asphalt−30% RP (SA). After rinse with de−ionized water: (**m**) matrix asphalt (SA); (**n**) Asphalt−10% RP (SA); (**o**) Asphalt−20% RP (SA); (**p**) Asphalt−30% RP (SA).

The base binder was 90# asphalt (Changchun Municipal Co., Ltd., Panjin, China) with properties detailed in Table 1. Four kinds of asphalt were prepared: matrix asphalt, Asphalt-10% RP, Asphalt-20% RP and Asphalt-30% RP using 40-mesh rubber powder. The specimens were exposed to 7-day freeze–thaw cycles in both clean water and a commercial de-icing salt solution (Shanxi Qingxue Co., Ltd., Xianyang, China). Microstructural characterization was conducted using a Leica DM4M optical microscope (Leica DM4M, Wetzlar, Germany), with macroscale and microscale images of rubber particles and deicer crystals presented in Figure 1a,b. Corresponding performance metrics are shown in Figure 1c,d.

The modified asphalt was prepared through the following procedure. First, the matrix asphalt and crumb rubber were preheated at 150 °C for 2 h in an oven. The preheated rubber powder was then blended into melted asphalt using a glass rod. Subsequently, the mixture underwent high-shear mixing at 5000 r for 60 min in a thermostatically controlled oil bath maintained at 150 °C. This process ensured homogeneous dispersion of crumb rubber powder throughout the asphalt binder. For the freeze–thaw experiments, the modified asphalt specimens were assigned to two groups: one immersed in clean water and the other in a 25 mass% de-icing salt solution (pH = 8.11). All specimens underwent seven freeze–thaw cycles at −20 °C, with each cycle lasting 24 h.

### 2.2. Microstructure Characterization

To characterize the microstructural evolution of rubber-modified asphalt under freeze–thaw conditions, OM and AFM (Bruker Multi Mode8, Wetzlar, Germany) were applied. The optical microscopy system used a condenser lens to focus light beams onto specimen surfaces. Reflected light was magnified through objective lenses to generate high-resolution images. AFM characterization with nanoscale resolution quantified morphological changes in bee structures between matrix asphalt and rubber-modified asphalt. Both techniques form a foundation for analyzing the microstructure and mechanical properties of matrix asphalt and modified asphalt.

### 2.3. FTIR Test

FTIR (Nicolet is50, Waltham, MA, USA) was employed to analyze functional group changes in asphalt after freeze–thaw cycles in clean water and the de-icing salt solution. Chemical characterization was performed using a Fourier-transform infrared spectrometer outfitted with a diamond attenuated total reflection (ATR) accessory. The ATR technique achieved molecular-level contact between a germanium crystal and specimen surfaces, detecting attenuated reflected light to capture surface vibrational information. Spectral acquisition parameters included a wavenumber range of 4000–650 cm^−1^, 32 accumulated scans, and 4 cm^−1^ spectral resolutions.

### 2.4. Temperature Sweep Test

Temperature sweep tests were conducted in triplicate following the AASHTO T 315-22 standard [26] to evaluate the dynamic viscoelastic properties of asphalt and two-way analysis of variance (ANOVA) was employed to investigate the main effects and interactive effects of crumb rubber content and environmental conditions on the viscoelastic behavior of asphalt binders. Samples were prepared using custom rubber molds with a diameter of approximately 17 mm and a depth of approximately 2 mm. Molten asphalt was poured into the mold, cooled to ambient temperature, and demolded. The specimens were then mounted in a dynamic shear rheometer equipped with 25 mm parallel plates, maintaining a 1 mm gap between plates. Testing was conducted across temperatures ranging from 46 °C to 64 °C in 6 °C intervals, with the frequency held constant at 10 rad/s. All test data were calculated and analyzed according to the following formulas.

(1) $$\tau =\frac{2T}{\pi r^{3}}$$

(2) $$\gamma =\frac{\theta r}{\pi r^{3}}$$

(3) $$G^{*}=\frac{\tau_{\max}-\tau_{\min}}{\gamma_{\max}-\gamma_{\min}}$$

(4) δ=2πf⋅Δt

where *T* is the maximum torque, *r* is the parallel plate radius, *θ* is the parallel plate rotation angle (rad), *h* is the height of the specimen, *τ_max_* is the maximum shear stress on the specimen, *τ_min_* is the minimum shear stress (kPa), *γ_max_* is the maximum shear strain, *γ_min_* is the minimum shear strain, *ƒ* is the parallel plate frequency (Hz), and Δ*t* is the strain lag time (s).

### 2.5. Multiple Stress Creep and Recovery Test

MSCR tests were performed in triplicate in accordance with AASHTO TP 70-13 [27] to evaluate the creep resistance and self-healing capacity of asphalt under complex loading conditions. The testing protocol applied two stress levels: 0.1 kPa for low-stress simulation and 3.2 kPa for high-stress simulation. Each stress level consisted of 10 consecutive cycles, with a 1 s loading phase followed by a 9 s recovery period. To assess the interactive influences of stress level (0.1 kPa and 3.2 kPa) and rubber content on viscoelastic parameters, two-way ANOVA was performed to examine significant differences in non-recoverable creep compliance (*J_nr_*) and recovery (*R*), with main effects and interaction terms evaluated via F-statistics and *p*-values. Rheological parameters, including non-recoverable creep compliance (*J_nr_*) and percent recovery (*R*), were calculated to quantify the viscoelastic behavior of the asphalt. The equations are presented below:

(5) $$J_{nr}=\frac{\gamma_{nr}}{\tau }$$

(6) $$R=\frac{\gamma_{p}-\gamma_{nr}}{\gamma_{p}-\gamma_{0}}$$

where *γ_nr_* is the residual strain within each loading cycle, *τ* is applied loading stress, *γ_p_* is the peak strain within each loading cycle, and *γ_0_* is the initial strain within each loading cycle.

### 2.6. Linear Amplitude Sweep Test

A Linear Amplitude Sweep testing was performed in accordance with AASHTO TP 101-21 [28] to characterize the fatigue response of asphalt. Temperature sweeps from 40 °C to 70 °C and frequency sweeps from 0.2 Hz to 30 Hz were conducted. Viscoelastic parameters and fatigue failure curves were initially obtained through frequency–domain analysis. The Williams–Landel–Ferry (WLF) equation was utilized to normalize rheological data via the application of the time–temperature superposition principle. Master curves were subsequently constructe, with the shift factor *α* calculated to quantify the rheological property of asphalt. The specific formula is as follows:

(7) $$\mathrm{lg}\alpha =-\frac{C_{1}\left(T-T_{0}\right)}{C_{2}+\left(T-T_{0}\right)}$$

where *C_1_* and *C_2_* are module parameters, and *T_0_* is the reference temperature.

## 3. Results and Discussion

### 3.1. Effect of Different Environments on Rubber-Modified Asphalt

**Figure 2:**
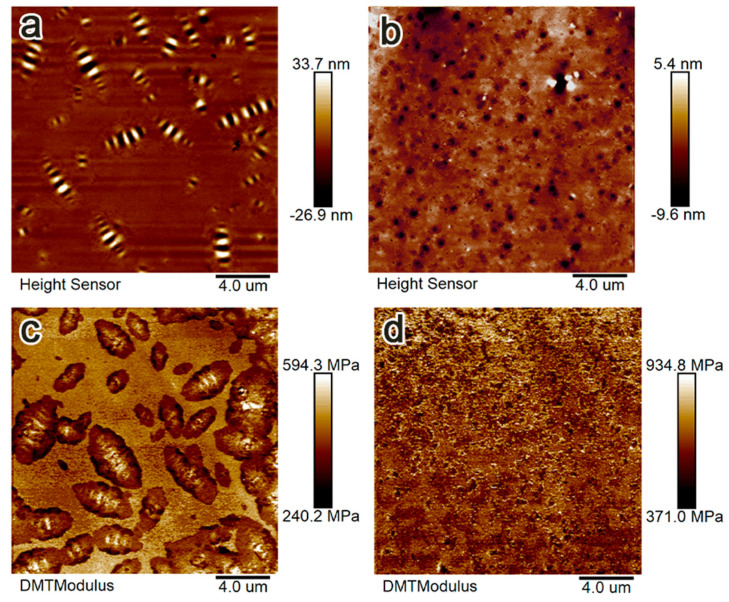
Microstructure of asphalt before freeze−thaw cycling on AFM: (**a**) 2D morphology of matrix asphalt; (**b**) 2D morphology of Asphalt−30% RP; (**c**) Young’s modulus image of matrix asphalt; (**d**) Young’s modulus image of Asphalt−30% RP.

The macroscopic and microscopic morphologies of crumb rubber and de-icing salts are presented in Figure 1a,d. AFM revealed that crumb rubber incorporation significantly increased the density of bee structures in asphalt (Figure 2). This microstructural modification led to a 33.59% increase in Young’s modulus for the Asphalt-30% RP compared to the matrix asphalt. The asphalt of improved modulus directly correlates with enhanced deformation resistance, inspiring further investigation into the performance of modified asphalt in harsh environmental scenarios.

Figure 1e–h illustrates the microstructural evolution of asphalt after one week of freeze–thaw cycling in clean water. Spherical yellow exudates were observed on the asphalt surface (Figure 1e), accompanied by an oil film on the water phase. This phenomenon is attributed to colloidal destabilization, migration of light components, and subsequent asphaltene enrichment. These processes are associated with the potential proliferation of bee structures [24,25,29,30]. With increasing crumb rubber content, light-component precipitation intensified, forming larger spherical aggregates (Figure 1f–h), a compositional shift that may facilitate denser bee structure formation. The observed morphological alterations, combined with findings from existing research [24,25,29,30], demonstrate a positive correlation between the proportion of heavy constituents and asphalt stability. These microstructural modifications fundamentally explain the reasons for mechanical improvement in rubber-modified asphalt under freeze–thaw conditions.

After de-icing salt-induced freeze–thaw cycles, a white crystalline layer was formed on the asphalt surface (Figure 1i–l). Optical microscopy analysis revealed this layer to be recrystallized de-icing salt. Polyhedral salt crystals were uniformly embedded within the asphalt matrix, partially penetrating its internal structure. This morphological configuration establishes itself as a mechanical interlocking mechanism, known as the pinning effect, wherein crystals act as physical anchors that restrict the mobility of asphalt molecular chains and impede microcrack propagation.

### 3.2. FTIR Analysis

**Figure 3:**
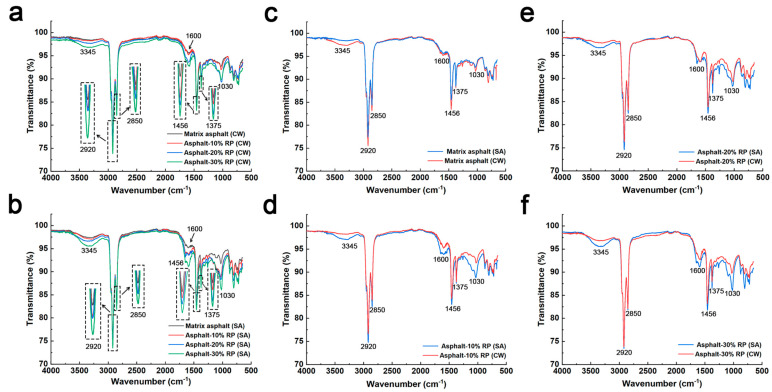
Infrared spectral analysis of freeze−thaw cycle asphalt of clean water and de−icing salt: (**a**) Spectra of different asphalts under clean water freeze−thaw cycles; (**b**) Spectra of different asphalts under de-icing salt freeze−thaw cycles; (**c**) Spectra of Matrix asphalt under different environments; (**d**) Spectra of Asphalt−10% RP under different environments; (**e**) Spectra of Asphalt−20% RP under different environments; (**f**) Spectra of Asphalt−30% RP under different environments.

Figure 3a,b present Fourier-transform infrared spectra comparisons between unmodified asphalt and crumb-rubber-modified asphalt after one-week freeze–thaw cycles in clean water and de-icing solutions. The characteristic peaks of both materials align similar, with no new functional groups observed, indicating no substantial chemical reactions between asphalt and crumb rubber. In both clean water and de-icing environments, the absorption intensities of methylene groups (2920 cm^−1^ and 2850 cm^−1^) and methyl groups (1456 cm^−1^ and 1375 cm^−1^) increase with higher crumb rubber contents [16,17]. This phenomenon stems from the conversion of light asphalt fractions into heavy components, thereby improving the asphalt’s high-temperature rutting resistance. At the C=C double bond region (1600 cm^−1^), rubber-modified asphalt exhibits stronger absorption than matrix asphalt. The stretching vibration peaks become more pronounced with increasing crumb rubber content, indicating improved deformation resistance [25]. Additionally, the absorption peak of hydroxyl groups (-OH) near 3345 cm^−1^ intensifies as crumb rubber content rises. This suggests that crumb rubber incorporation strengthens intermolecular hydrogen bonds, hinders molecular mobility, and increases asphalt stability [31].

Within the characteristic absorption bands of methylene (2920 cm^−1^ and 2850 cm^−1^) and methyl groups (1456 cm^−1^ and 1375 cm^−1^), rubber-modified asphalt specimens subjected to de-icing salt freeze–thaw cycles displayed significantly increased peak intensities compared to those exposed to clean water freeze–thaw cycles (Figure 3c–f). This increase results from the reaction of short-chain hydrocarbons to form longer-chain compounds, enhancing resistance to permanent deformation [32]. At hydroxyl groups (3345 cm^−1^), asphalt subjected to de-icing salt exhibits intensified absorption peaks, attributed to the salting-out effect and ionic interactions. Similarly, intensified stretching vibrations of sulfoxide (1030 cm^−1^) and carbonyl (1600 cm^−1^) groups were observed. While no macroscopic molecular structural alterations were detected, these spectral enhancements confirm the transformation from light to heavy components, thereby elevating intermolecular cohesion [22,24,33]. This molecular stabilization arises from strengthened dipole–dipole interactions, driven by the high dipole moments of sulfoxide and carbonyl functional groups. These polar moieties generate robust electrostatic networks that enhance intermolecular binding energy, effectively inhibiting molecular chain slippage and consequently improving deformation resistance. Matrix asphalt subjected to de-icing salt freeze–thaw cycles exhibited weaker absorption peaks near sulfoxide, carbonyl, hydroxyl, methylene, and methyl groups compared to specimens subjected to clean water freeze–thaw cycles. This indicates that de-icing salt can erode the base asphalt, break chemical bonds or intermolecular forces. In contrast, crumb-rubber modification enhances asphalt’s resistance to de-icing salt erosion and improves structural stability. These findings offer a theoretical foundation for the engineering application of rubber-modified asphalt in cold regions.

### 3.3. The Rutting Resistance

**Figure 4:**
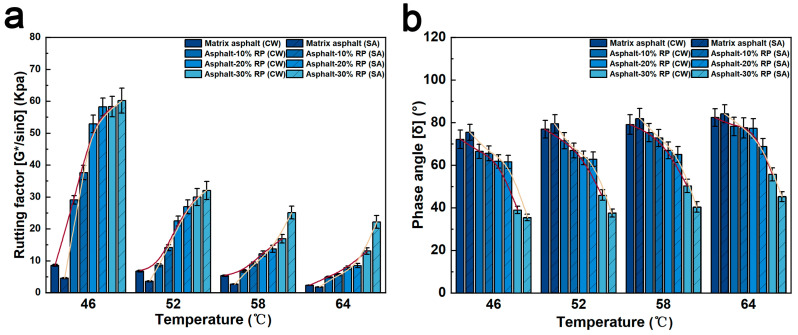
(**a**) Rutting factor of modified asphalts under different environments; (**b**) Phase angle of modified asphalts under different environments.

The high-temperature resistance to rutting can be characterized by indicators such as rutting factor and phase angle. Figure 4a illustrates the rutting factor evolution of matrix asphalt and modified asphalt under clean water and de-icing salt freeze–thaw cycles, with two-way ANOVA confirming significant main effects (rubber content: F = 386.2, *p* < 0.0001; environment: F = 19.5, *p* = 0.0002). The rutting factor increases with crumb rubber content at identical temperatures, with specimens under de-icing salt solutions consistently outperforming those in clean water despite non-significant interaction (F = 1.34, *p* = 0.287). At 46 °C, in the environments of clean water and de-icing salt solutions, the rutting factors of Asphalt-30% RP increased by 49.75 kPa and 55.66 kPa, respectively, compared with the matrix asphalt, demonstrating rubber’s dominant effect. This can be attributed to the enhanced stability resulting from rubber incorporation. In contrast, the rutting factors of matrix asphalt subjected to de-icing salt freeze–thaw cycles were lower than those under clean water freeze–thaw conditions, presumably due to structural degradation caused by NaCl and CH_3_COONa. Rubber-modified asphalt, however, undergoes chemical interactions with de-icing salt, enhancing the content of sulfoxide (S=O) and carbonyl (C=O) groups (Figure 2). These oxygenated functional groups strengthen intermolecular interactions, enabling superior post-freeze–thaw mechanical performance and validating the efficacy of crumb rubber in enhancing the resistance of asphalt to erosion by de-icing salt freeze–thaw cycles.

Figure 4b systematically examines phase angle variations of asphalt binders across temperature gradients, where ANOVA revealed phase angle sensitivity (rubber: F = 345.2, *p* < 0.0001; environment: F = 58.5, *p* < 0.0001; interaction: F = 35.8, *p* < 0.0001). A temperature increase from 46 °C to 64 °C raises the phase angle by approximately 7 degrees, suggesting that temperature acts as the primary driver of viscoelastic behavior evolution. Elevated temperatures accelerate molecular mobility in asphalt, thereby decreasing viscosity and causing an increase in the phase angle. Specifically, Asphalt-30% RP exhibits a 3.58° differential at 46 °C, escalating to 10.5° at 64 °C, confirming that temperature amplifies interaction effects. This suggests that elevated temperatures intensify the interactions between de-icing salt and rubber-modified asphalt, and it also indicates that the material can retain stable performance after exposure to harsh environments.

Overall, crumb rubber addition significantly affects the rutting factors and phase angles of asphalt under varying freeze–thaw cycles, enhancing its high-temperature rutting resistance and post-harsh-environment stability. However, designing de-icing salt-resistant asphalt pavements requires not only an evaluation of high-temperature rutting resistance but also a comprehensive consideration of other critical mechanical properties.

### 3.4. The Creep Resistance

**Figure 5:**
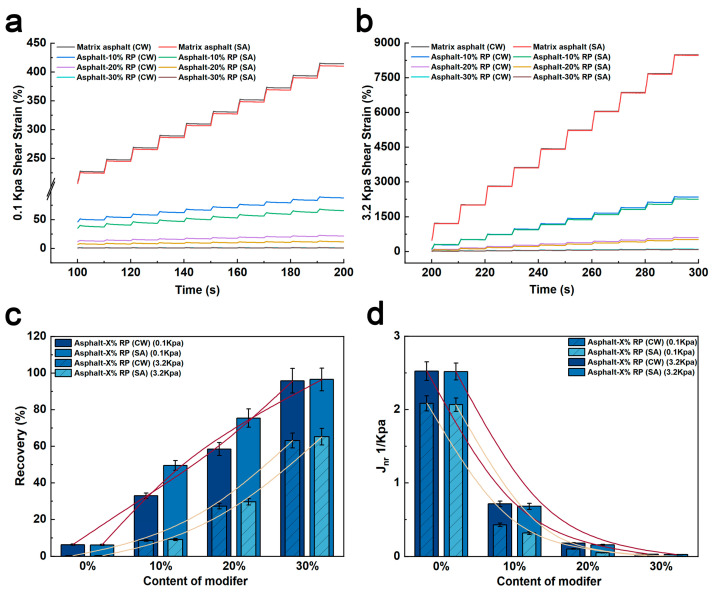
(**a**) Shear strain at 0.1 kPa; (**b**) shear strain at 3.2 kPa; (**c**) Recovery of modified asphalts under different environments; (**d**) *J_nr_* of modified asphalts under different environments.

Figure 5a,b compare the creep deformation evolution of matrix asphalt and rubber-modified asphalt under clean water and de-icing salt freeze–thaw cycles. At 0.1 kPa loaded for 200 s, the Asphalt-30% RP exhibited shear strain 413.21% and 409.35% lower than matrix asphalt in clean water and de-icing salt environments, respectively. The incorporation of crumb rubber enhances the absorption peaks of C=C double bonds and methyl groups, promoting the formation of heavy components in asphalt and improving its deformation resistance [22,33].

Figure 5c compares the creep recovery rates of rubber-modified asphalt in clean water and de-icing salt environments. Under 0.1 kPa loading, the matrix asphalt subjected to de-icing salt freeze–thaw cycles exhibited a creep recovery rate 2.8% lower than that of specimens subjected to clean water freeze–thaw cycles. In contrast, Asphalt-10% RP and Asphalt-20% RP exhibited significantly higher creep recovery rates in de-icing salt environments—50.53% and 28.94% higher, respectively, than in clean water freeze–thaw cycles. Notably, Asphalt-30% RP exhibited negligible performance variation across environments, with a recovery rate difference of only 0.75%. The experimental results demonstrate that crumb rubber effectively mitigates the adverse effects of de-icing salt on the creep recovery behavior of asphalt. Notably, within a specific crumb rubber content range, the creep recovery performance of rubber-modified asphalt in de-icing salt environments first improves and then stabilizes as the rubber content increases. Two-way ANOVA confirmed this trend with significant interaction (F = 144.1, *p* < 0.0001), where salt exposure improved recovery by 50.53% in Asphalt-10% RP and 28.94% in Asphalt-20% RP. This elastic recovery enhancement complements the inverse correlation of non-recoverable creep compliance (J_nr_), a critical indicator of viscous behavior, and exhibits an inverse correlation with high-temperature rutting resistance, where lower J_nr_ values indicate superior rutting performance. Figure 5d shows that J_nr_ decreases progressively with increasing crumb rubber content under both 0.1 kPa and 3.2 kPa stress levels, confirming the enhanced rutting resistance of rubber-modified asphalt as validated by ANOVA (0.1 kPa: F = 382.5, *p* < 0.0001; 3.2 kPa: F = 385.2, *p* < 0.0001). Notably, under 3.2 kPa loading, asphalt specimens subjected to de-icing salt freeze–thaw cycles exhibited lower J_nr_ than those subjected to clean water freeze–thaw cycles, corroborated by significant main effects (rubber content: F = 385.2, *p* < 0.0001; environment: F = 24.7, *p* = 0.0001), though interaction was non-significant (F = 2.85, *p* = 0.118). The percentage differences in J_nr_ followed the following order: Asphalt-10% RP > Asphalt-20% RP > matrix asphalt > Asphalt-30% RP. Despite the non-significant interaction (F = 2.85, *p* = 0.118), the robust main effect of de-icing salt (F = 19.6, *p* = 0.0003) indicates that salt-induced J_nr_ reduction occurs consistently across rubber-modified systems, though the magnitude varies by different rubber powder contents. The percentage difference between matrix asphalt and Asphalt-30% RP in both environments was less than 1% (*p* > 0.05). These results indicate that moderate crumb rubber contents significantly improve resistance to de-icing salt erosion. From a mechanistic perspective, de-icing salt induces oxidative crosslinking reactions in rubber-modified asphalt, promoting the formation of stabilized molecular structure.

The synergistic improvement in elastic recovery and non-recoverable creep reduction demonstrates crumb rubber’s dual-phase enhancement mechanism, which involves both elastic reinforcement and viscous reduction. In summary, crumb rubber modification significantly enhances the performance of asphalt under multiple environmental conditions. The incorporation of crumb rubber improves structural stability after both clean water freeze–thaw cycles and de-icing salt exposure. In actual engineering, it is necessary to determine the optimal dosage to achieve the maximum economic benefits and mechanical properties.

### 3.5. The Fatigue Resistance

**Figure 6:**
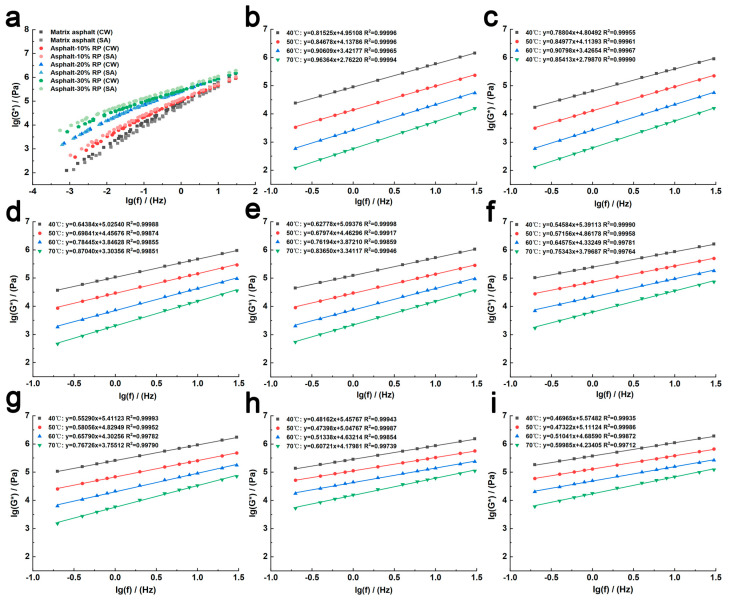
(**a**) Complex modulus master curves of asphalt under different environments; (**b**) complex modulus master curves of matrix asphalt (CW); (**c**) complex modulus master curves of matrix asphalt (SA); (**d**) complex modulus master curves of Asphalt−10% RP (CW); (**e**) complex modulus master curves of Asphalt−10% RP (SA); (**f**) complex modulus master curves of Asphalt−20% RP (CW); (**g**) complex modulus master curves of Asphalt−20% RP (SA); (**h**) complex modulus master curves of Asphalt−30% RP (CW); (**i**) complex modulus master curves of Asphalt−30% RP (SA).

Figure 6b–i show the influence of frequency on the complex shear modulus of modified asphalt across four temperature conditions (40 °C, 50 °C, 60 °C, and 70 °C), with both modulus and frequency plotted on logarithmic scales. Figure 6a shows the frequency-dependent complex shear modulus master curve for various asphalts at the reference temperature of 40 °C. The data exhibits a consistent trend: at constant temperatures, complex shear modulus increases with rising frequency. For instance, at 70 °C, increasing the frequency from 0.2 Hz to 30 Hz caused the Asphalt-30% RP to exhibit complex shear modulus increases of 35.85% (clean water freeze–thaw) and 34.39% (de-icing salt freeze–thaw). These increments are 35.58% and 37.91% lower than those of matrix asphalt, respectively. The complex shear modulus growth in rubber-modified asphalt indicates more gradual responses to frequency variations, signifying that crumb rubber incorporation enhances asphalt stability under varying loading frequencies and reduces its susceptibility to fatigue failure.

As a viscoelastic material, asphalt demonstrates strong adherence to the time–temperature superposition principle. Using the time–temperature superposition principle with calculated shift factors, a master curve of complex shear modulus referenced to 40 °C was constructed. The results showed that the shift factors of rubber-modified asphalt, calculated using MATLAB software (R2020a), exhibited a strong correlation with the WLF equation (R^2^ > 0.8), as listed in Table 2. Figure 6 illustrates that the complex shear modulus of modified asphalt increases progressively with increasing crumb rubber content. At equivalent crumb rubber dosages, asphalt subjected to de-icing salt freeze–thaw cycles demonstrates higher complex shear modulus values than those treated with clean water freeze–thaw cycles. In contrast, matrix asphalt exhibits smaller complex shear modulus under de-icing salt conditions. This divergence highlights the enhancement mechanisms of crumb rubber: chemical interactions with de-icing salt promote the formation of sulfoxide (S=O) and carbonyl (C=O) groups, increasing heavy components to improve deformation resistance [22,24,33]. In addition, the addition of rubber powder can increase the density of bee structures in asphalt (Figure 2) and enhance the stability of asphalt [30].

In summary, crumb rubber powder notably enhances the mechanical properties of asphalt under both de-icing salt and clean water freeze–thaw regimes, thereby establishing a theoretical basis for cold-region asphalt-based pavement designs.

## 4. Conclusions

(1) Clean water freeze–thaw cycles promote the precipitation of oil components within asphalt, increasing the density of bee structures microstructures and enhancing asphalt stability. When asphalt undergoes de-icing salt freeze–thaw cycles, the de-icing salt recrystallizes on the asphalt surface and leads to pinning effect with the asphalt. This phenomenon induces and strengthens ion–dipole interactions between salt ions and asphalt molecules. As a result, by inhibiting molecular mobility, the structural stability of asphalt is enhanced.
(2) Fourier transform infrared spectroscopy shows intensified absorption peaks of methyl (CH_3_) and carbonyl (C=O) groups in rubber-modified asphalt after freeze–thaw cycles under water and de-icing salt conditions. Rubber-modified asphalt exhibits stronger peaks under de-icing salt environments than under clean water conditions. These changes increase the content of heavy components in asphalt and enhance its deformation resistance.
(3) Dynamic shear rheological analysis reveals that crumb-rubber modification significantly improves the viscoelastic behavior of asphalt binders. Rubber-modified asphalt under de-icing salt freeze–thaw cycles demonstrates superior mechanical performance. Creep recovery analysis reveals that Asphalt-10% RP and Asphalt-20% RP exhibit recovery rates 50.53% and 28.94% higher, respectively, under de-icing salt freeze–thaw conditions compared to those under clean water freeze–thaw conditions.

## Figures and Tables

**Table 1:** Performance indexes of matrix asphalt.

Item	Actual Measurement	Standard Requirement
Penetration (100 g, 5 s, 0.1 mm)	85	80~100
Softening point (°C)	46.3	≥42
Ductility (cm)	>100	≥100
Viscosity (cp)	340	——

**Table 2:** Displacement factors of rubber powder-modified asphalt.

Category	Displacement Temperature				WLF Equation Fitting		
	40 °C	50 °C	60 °C	70 °C	C1	C2	R2
Matrix asphalt (CW)	0	−0.95813	−1.68179	−2.26223	7.13398	64.66532	0.99997
Matrix asphalt (SA)	0	−0.79517	−1.48537	−2.32969	5.91873	54.82843	0.99966
Asphalt-10% RP (CW)	0	−0.81727	−1.51019	−1.98848	6.52214	67.87463	0.99921
Asphalt-10% RP (SA)	0	−0.93942	−1.62965	−2.13241	5.78238	51.24426	0.99996
Asphalt-20% RP (CW)	0	−0.9584	−1.75027	−2.31344	7.48393	66.65235	0.99954
Asphalt-20% RP (SA)	0	−1.03747	−1.80387	−2.36627	6.51555	52.50572	0.99996
Asphalt-30% RP (CW)	0	−0.8497	−1.66682	−2.30102	12.96199	138.21525	0.99923
Asphalt-30% RP (SA)	0	−0.98886	−1.83932	−2.50083	10.05768	90.34118	0.99979

## Data Availability

The testing and analysis data used to support the findings of this study are included within the article.
